# BSmooth: from whole genome bisulfite sequencing reads to differentially methylated regions

**DOI:** 10.1186/gb-2012-13-10-r83

**Published:** 2012-10-03

**Authors:** Kasper D Hansen, Benjamin Langmead, Rafael A Irizarry

**Affiliations:** 1 Department of Biostatistics, Johns Hopkins Bloomberg School of Public Health, Baltimore, MD 21205, USA; 2 Center for Epigenetics, Johns Hopkins University School of Medicine, Baltimore, MD 21205, USA

## Abstract

DNA methylation is an important epigenetic modification involved in gene regulation, which can now be measured using whole-genome bisulfite sequencing. However, cost, complexity of the data, and lack of comprehensive analytical tools are major challenges that keep this technology from becoming widely applied. Here we present BSmooth, an alignment, quality control and analysis pipeline that provides accurate and precise results even with low coverage data, appropriately handling biological replicates. BSmooth is open source software, and can be downloaded from http://rafalab.jhsph.edu/bsmooth.

## Background

DNA methylation is an important epigenetic modification involved in gene silencing, tissue differentiation, and cancer [1]. High-resolution, genome-wide measurement of DNA methylation is now possible using whole-genome bisulfite sequencing (WGBS), a process whereby input DNA is treated with sodium bisulfite and sequenced. While WGBS is comprehensive, it is also quite costly [2]. For instance, an application of WGBS by Lister *et al*. [3] compared DNA methylation profiles of an embryonic stem cell line and a fibroblast cell line. Both were sequenced to about 30× coverage (25× coverage of all CpGs), requiring 376 total lanes of bisulfite sequencing on the Illumina GA II instrument. While conventional wisdom is that 30× coverage or deeper is needed to achieve accurate results, advanced statistical techniques proposed here, such as local likelihood smoothing, can reduce this requirement to as little as 4×.

**Figure 1:**
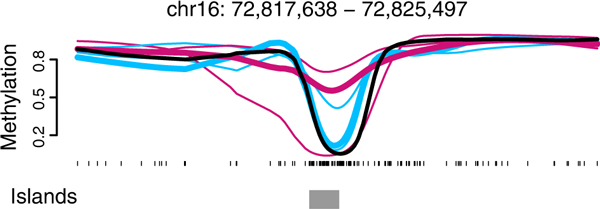
**The need for biological replicates**. We show smoothed methylation profiles for three normal samples (blue) and matched cancers (red) from the Hansen data [1]. Also shown is the smoothed methylation profile for an IMR90 cell-line (black) from the Lister data [3]. Had we only analyzed normal-cancer pair 3 (thick lines), there would appear to be a methylation difference between cancer and normal in this genomic region. When all three cancer-normal pairs are considered, however, this region does not appear to be a cancer-specific differentially methylated region.

It has also been shown that different genomic regions exhibit different levels of DNA methylation variation among individuals [4]. As a consequence, regions that are inherently variable can easily be confused with regions that differ consistently between groups when few replicates are available [1] (Figure 1). But performing WGBS on the number of biological replicates required to overcome such issues can be quite expensive. The techniques proposed here address this issue both by making full use of replicate information during analysis, and by potentially reducing the coverage needed for (and therefore the cost of) replication.

Analysis of WGBS data starts with alignment of bisulfite converted reads. After alignment, statistical methods are employed to identify differentially methylated regions (DMRs) between two or more conditions. Extensive work has been dedicated to alignment [5-10] but methods for post-alignment analysis are limited. Published work based on WGBS has relied on a modular approach that first identifies differentially methylated CpGs that are then grouped into regions using *ad hoc *grouping rules. The first step is carried out using either Fisher's exact test [3,11-13], arbitrary cutoffs for differences in observed methylation levels [14], or a beta-binomial model [15]. None of these methods take biological variability into account. To the best of our knowledge, no software is available implementing these approaches.

Here we present BSmooth, a comprehensive analysis tool for WGBS datasets. The BSmooth pipeline begins with an unbiased and bisulfite-aware read alignment step, compiles quality assessment metrics based on stratifying methylation estimates by read position, applies local averaging to improve precision of regional methylation measurements, and detects DMRs accounting for biological variability when replicates are available. The main methodological contribution of BSmooth is the ability to identify DMRs accounting for biological variability, as well as the quality control measures we propose. In addition, BSmooth includes a new aligner, Merman, which appropriately handles colorspace. We demonstrate the benefits of BSmooth with four publicly available datasets: the Lister data [3], the Hansen data [1], the Hansen-capture data [1] and the Tung data [16] (see Materials and methods for details). We use these data to demonstrate the advantages of BSmooth over existing algorithms based on Fisher's exact test. BSmooth is the first pipeline for WGBS datasets yielding DMRs as output, while also taking biological variation into account. It can handle low-coverage experimental designs, allowing researchers to profile several samples at the same cost as a high-coverage profile of a single sample.

## Results and discussion

### Alignment

Sodium bisulfite treatment converts unmethylated cytosine (C) nucleotides to uracils, which are reported as thymines (T) by the sequencer, and leaves methylated cytosines unmodified. When sequencing reads derived from treated DNA are aligned to a reference genome, the methylation status of a C in the reference can be measured by examining aligned reads overlapping it. For instance, when a C in a bisulfite-treated read overlaps a C in the reference, this indicates the reference C is methylated in at least one molecule in the sample.

Alignment of sequencing reads derived from bisulfite-treated DNA is complicated by the fact that a reference C's methylation status affects the scores of alignments covering it. This can result in bias either toward or against alignments covering methylated cytosines. Algorithms have been proposed that avoid bias by removing the penalty associated with aligning a C or T in the read to a C in the reference genome. One such approach is '*in silico *bisulfite conversion', whereby C nucleotides both in the reads and in the reference genome are converted to T nucleotides prior to alignment [3,8]. A related approach is to convert only the reference genome in this way [17,18], but this results in bias against reads overlapping both methylated and unmethylated cytosines.

Other approaches avoid bias by, at some point in the alignment process, considering all possible combinations of methylation status. VerJinxer [5] and BSMAP [9], for example, build a 'seed' index of the reference genome. For each seed extracted, multiple versions of the seed are added to the index: one for each possible assignment of either C or T to a position that originally contained a C. This ensures that the index-assisted alignment steps are not biased by methylation status. The approach of PASH [6] is similar, with seeds being extracted from the read rather than the reference.

An advantage of *in silico *bisulfite conversion is that post-conversion alignment can be performed using a fast tool such as Bowtie [19]. A disadvantage is that it does not straightforwardly handle 'colorspace' reads from the SOLiD sequencing instrument. For this reason, BSmooth implements two alignment algorithms, which the user may choose between. The first is based on *in silico *bisulfite conversion and uses Bowtie 2 [20] to align. Because it uses Bowtie 2, this pipeline fully supports gapped alignment and alignment of paired-end bisulfite-treated reads. The second pipeline uses a new aligner called Merman, which supports unbiased alignment of colorspace bisulfite reads. Merman extends the indexing approach of VerJinxer [5] and BSMAP [9]; as in those approaches, we build a 'seed' index of the reference genome. Instead of extracting nucleotide subsequences, though, we extract corresponding color subsequences. For each subsequence extracted, multiple versions may be added to the index: one for each color subsequence resulting from each possible assignment of either C or T to positions originally containing a C. This closely follows the approaches of VerJinxer [5] and BSMAP [9], but additionally translates nucleotide ambiguity into color ambiguity. The Merman-based pipeline does not support gapped alignment or paired-end alignment.

The Merman alignment pipeline is included chiefly to allow users to align a greater breadth of input types. It is generally slower and less memory-efficient than the Bowtie 2-based pipeline. BSmooth also allows the user to bypass the alignment stage, in which case the user must provide a collection of SAM [21] files formatted as though they had been generated by one of BSmooth's pipelines. A comparison between different alignment strategies demonstrated that the effect on downstream results is negligible (Figure S5 in Additional file 1), compared to technical variation. As we demonstrate below, the choice of statistical analysis method has a much stronger impact.

### Quality control

**Figure 2:**
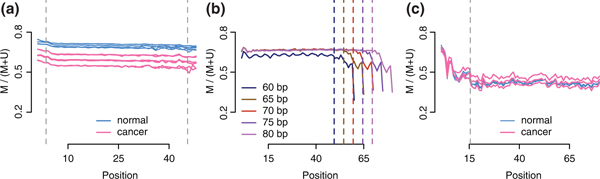
**Quality control plots**. **(a) **M-bias plot for the Hansen data, a WGBS experiment on cancer samples. Each sample was sequenced on two flowcells. We show the methylation proportion across each possible read position. This plot shows limited evidence of methylation bias across the read positions. Vertical lines indicate cutoffs used for M-bias filtering. **(b) **M-bias plots for the Lister data, a WGBS experiment in a fibroblast cell line. These data were aligned using iterative trimming and each read length is depicted separately (different colors). The plot shows methylation bias toward the end of reads for all read lengths. **(c) **M-bias plot for the Hansen-capture data, a capture bisulfite sequencing experiment on cancer samples. The plot shows methylation bias at the start of the reads.

Systematic sequencing and base-calling errors that adversely affect downstream results are common and increasingly well characterized [22,23]. For instance, incorrect base calls toward the 3' ends of reads can favor specific nucleotides [23]. We observed similar biases in WGBS data and developed a sample-specific quality assessment plot to visualize them. For each uniquely aligned read, we recorded read positions corresponding to CpG cytosines in the reference, along with the read base overlapping that position if it is C (methylated) or T (unmethylated). We refer to these as the read-level measurements. We then stratified these measurements by read position, computed the percent of Cs in each stratum, and plotted them (Figure 2). Since methylation state should not depend on read position, these plots ought to show a flat horizontal line (Figure 2a). However, biases were observed in two of the three examined datasets (Figure 2b,c). We therefore refer to them as M-bias plots. For datasets with mixed read lengths, we recommend one plot per read length (Figure 2b; Figures S1 to S3 in Additional file 1).

These plots can also be used to make filtering decisions. In the three datasets we examined, inspection of the M-bias plot motivated restricting the read positions used to a certain range: read-level measurements for which the position was outside this range were excluded from further analysis (but the whole read was still used for alignment). We refer to this procedure as M-bias filtering. In the Lister data we excluded the last 10 bp from each trimmed read. In the Hansen dataset we excluded measurements from the first three and last three positions (Figure 2a). In the Hansen-capture dataset we excluded measurements from the first 15 positions (Figure 2c). This filtering led to substantially increased agreement between the datasets representing the same sample processed with two different protocols (Figure S4 in Additional file 1).

### Smoothing

**Figure 3:**
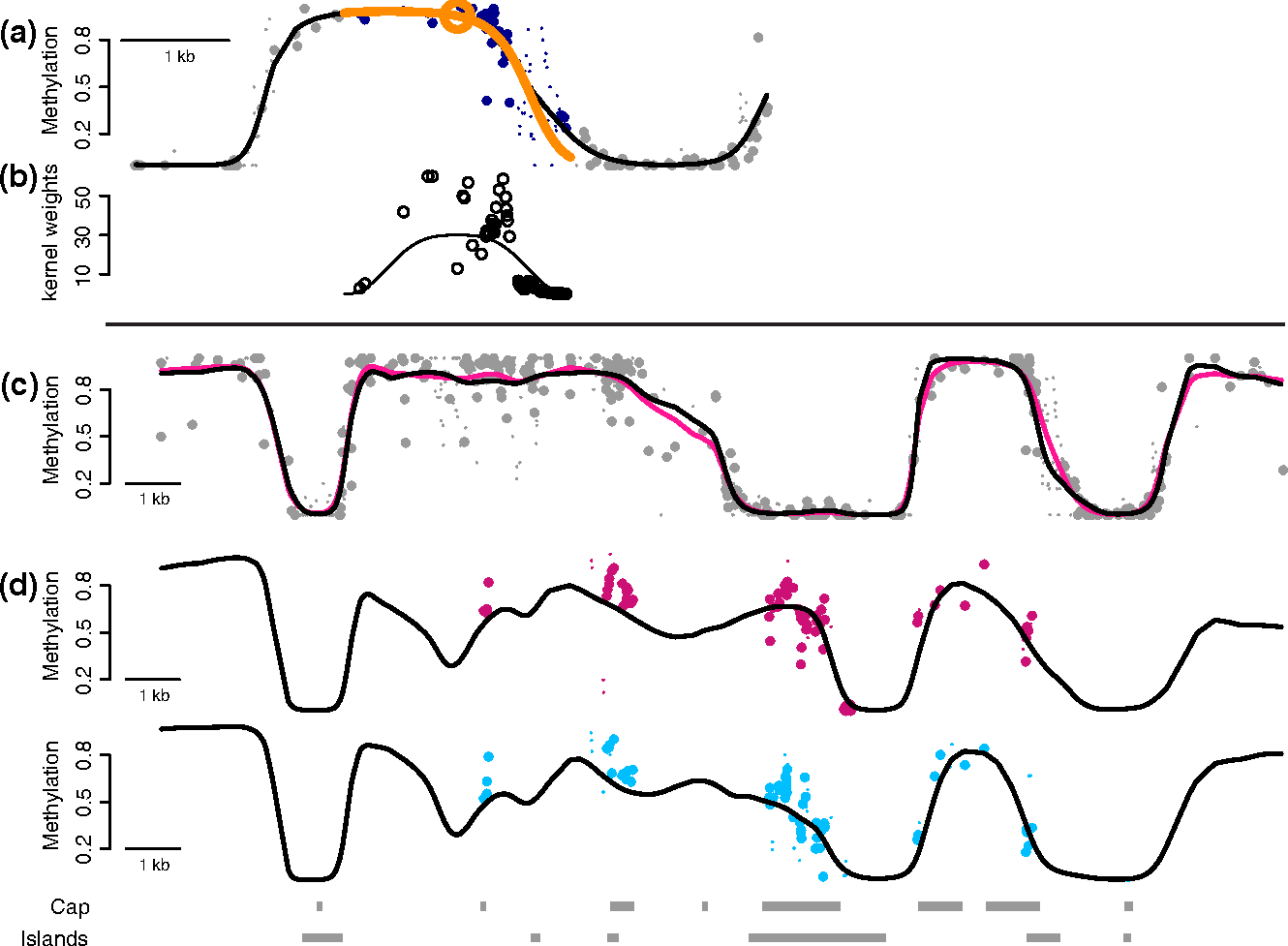
**The advantages of smoothing**. **(a) **Points represent single-CpG methylation estimates plotted against their genomic location. Large points are based on greater than 20× coverage. The orange circle denotes the location for which we are estimating the methylation profile. The blue points are those receiving positive weight in the local likelihood estimation. The orange line is obtained from the fitted parabola. The black line is the methylation profile resulting from repeating the procedure for each location. **(b) **The curve represents the kernel used in the weighted regression and the points are the actual weights, which are also influenced by coverage. **(c) **Points are as in (a) for the 25× coverage Lister data. The pink line is obtained by applying BSmooth to a the full data. The black line is the estimate from BSmooth based on a 5× subset of the Lister data. **(d) **The points are as in (a) but for the Hansen-capture data with average 35× coverage, and average across three replicates. The black line is the BSmooth estimate obtained from the 4× Hansen data, averaged across three replicates.

We employed smoothing to estimate the methylation level in a genomic region for a single sample. We denote the number of reads associated with the *j*th CpG being methylated and unmethylated with *M_j _*and *U_j _*respectively. The CpG-level summary is simply the proportion *M_j_*/*N_j_*, with *N_j_*=*M_j_*+*U_J _*the coverage for the *j*th CpG. We assume each *M_j _*follows a binomial distribution with success probability π*_j_*. The success probability represents the true proportion of chromosomes for which the *j*th CpG is methylated in the sample being assayed. The proportion *M_j_*/*N_j_*, denoted the single-CpG methylation estimate, is an unbiased estimate of π*_j _*with standard error $\sqrt{\pi_{j}\left(1-\pi_{j}\right)N_{j}}$. This has led most WGBS studies to employ a high coverage design since even 30× coverage yields standard errors as large as 0.09. However, various authors have noted that methylation levels are strongly correlated across the genome [24,25]. Furthermore, functionally relevant findings are generally associated with genomic regions rather than single CpGs, either CpG islands [26], CpG island shores [27], genomic blocks [1], or generic 2 kb regions [3]. This implies that we can assume that π_j _varies smoothly along the genome, without distorting signal or losing functional information. We can therefore improve precision by the use of modern statistical techniques such as local likelihood smoothing [28] (see Materials and methods for details; Figure 3a,b).

Using this method on data with 4× coverage, we achieved precision comparable to deeper coverage without smoothing. Specifically, we applied BSmooth to a subset of the IMR90 cell line study with 5× coverage; we used one of the six different library preparations applied to two different DNA extractions [3]. We compared the estimated methylation profile based on the 5× data to results obtained using the full data; for each CpG we averaged single-CpG methylation estimates based on the full 30× data over a 1 kb interval using only loci with at least 10× coverage. We found close agreement between the two sets of results (Figure 3c) with a correlation of 0.90 and a median absolute difference of 0.056. Additionally, when smoothing both high coverage data and low coverage data there was also close agreement: correlation of 0.97 and a median absolute difference of 0.024, using all CpGs in the genome. These two results show that we accurately estimate regional methylation level using low coverage data, and that there is little difference between the results of smoothing a high coverage dataset and the results of smoothing a low coverage dataset.

We also compared low coverage colon cancer data to high coverage capture data obtained with padlock probes (Figure 3d). For the capture data we only considered CpGs with 30× coverage or greater and computed an average methylation level across each capture region. Using the smoothed methylation profiles, an average smoothed methylation level was computed by averaging the smoothed value for all CpGs in the capture region. We found excellent agreement, with correlations between 0.89 and 0.92 and median absolute differences between 0.045 and 0.069. Additionally, there is a striking qualitative agreement between the single-resolution CpG estimates from the high-coverage capture data and the results of smoothing the low-coverage WGBS data (Figure 3d). Note that the two datasets being compared here, unlike the IMR90 data described above, were generated using two very different protocols, performed in two separate laboratories.

### Differentially methylated regions

Previous publications have focused on precisely estimating methylation levels at single-base resolution. For example, Fisher's exact test has been used to identify CpGs differentially methylated across two samples [3]. However, these studies are ultimately concerned with DMRs or differences between groups of samples. For example, Lister *et al*. [3] searched for genomic regions containing many differentially methylated CpGs, resulting in DMRs that are at least 2 kb long. A problem with this approach is that Fisher's exact test accounts for DNA sampling variability but not biological variability. Biological variability is well-established [4] and necessitates biological replicates from each group under consideration [1]. The goal is then to find regions that exhibit consistent differences even when taking biological variation into account. The DMR detection algorithm implemented in BSmooth is based on a statistic that appropriately summarizes consistent differences. Briefly, we first use the local-likelihood approach to estimate a sample-specific methylation profile, then compute estimates of the mean differences and standard errors for each CpG to form a statistic similar to that used in a *t*-test (see Materials and methods for details).

**Figure 4:**
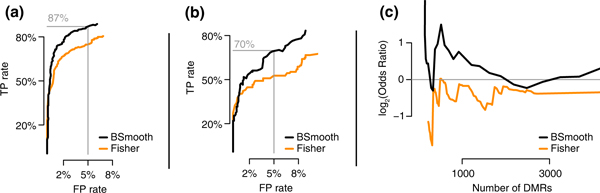
**Evaluation of the differentialy methylated regions finder**. **(a) **Specificity plotted against sensitivity for the BSmooth DMR finder (black) and a method based on Fisher's exact test (orange) applied to the Hansen data. The gold-standard definition is based on mean differences. Details are explained in the text. **(b) **As (a), but using a gold-standard definition accounting for biological variation. **(c) **Comparison based on the association between gene expression and methylation changes in the Tung data. For DMR lists of varying sizes (x-axis), the log2-odds ratios of finding a DMR within 5 kb of the transcription start site of a differentially expressed gene (FDR ≤5%) compared to genes not differentially expression (FDR ≥25%) are shown. FP, false positive; TP, true positive.

We applied BSmooth to identify DMRs between normal colon and colon cancer in the Hansen dataset. To address how well our method compares to having high-coverage data, we used the Hansen-capture bisulfite sequencing data as gold-standard and created receiver operating characteristic (ROC) curves. Specifically, we computed the average methylation difference between the cancer and normal samples inside each capture region, using only CpGs with 30× or more coverage, and considered this to be gold-standard measurements. We defined positives and negatives in two ways: one based on mean differences and the other taking biological variability into account. Specifically, for the first, we defined positives as capture regions with an average difference >0.25 using the gold-standard measurements (364 regions) and negatives as those with average differences <0.03 (2,012 regions; see Materials and Methods for details). This definition does not take biological variability into account. We computed false and true positive rates for different cutoff choices for the t-statistic by counting how many reported regions overlapped, by more than 50%, with gold standard positive and negative regions, respectively. We also required that the reported regions show methylation differences going in the same direction as the gold standard differences. Here, our method achieved 87% sensitivity at 95% specificity (Figure 4a). In the second definition of positives and negatives we accounted for biological variability by using a Welch *t*-test on the gold-standard measurements. Specifically, positives were defined as regions with an unadjusted *P*-value from the Welch *t*-test of <1% (114 regions) and negatives as those with an unadjusted *P*-value >25% (925 regions). Using this definition, our method achieved 70% sensitivity at 95% specificity (Figure 4b). We compared the results of BSmooth to the results of a method using Fisher's exact test [3] (see Materials and Methods for details). Because the Fisher based method does not account for biological variability, we pooled the data from the three cancer samples and the three normal samples. We compared the two methods using ROC curves and demonstrated that BSmooth outperforms the existing method (Figure 4a,b).

We also applied BSmooth to the Tung dataset. Tung *et al*. [16] studied the relationship between gene expression and social rank in a cohort of 49 monkeys, using microarrays. Out of the 6,097 genes studied, they identified 454 to be significantly related to social rank at a false discovery rate (FDR) of 5%. These 6,097 genes map to a total of 9,386 transcription start sites (TSSs). To assess the extent to which methylation might be involved in regulating the observed expression changes, they also performed WGBS on three high ranking and three low ranking individuals at medium CpG coverage (11× to 14×). Again, we compare the results of BSmooth to the results of a method using Fisher's exact test. Figure 4c depicts the log-odds ratio for finding a DMR near (within 5kb) the TSS of a differentially expressed gene (FDR ≤5%) compared to finding a DMR near the TSS of a gene not differentially expressed (FDR ≥25%). This figure shows that BSmooth consistently finds more DMRs near TSSs of differentially expressed genes compared to the method based on Fisher's exact test. We note that the odds ratio for Fisher's exact test is slightly below one, suggesting that this test is no better than random guessing at finding DMRs near differentially expressed genes. Due to the low percentage of differentially expressed genes, Fisher's exact test does not have enough sensitivity to detect the associated DMRs.

The code used for the results presented above are included as Additional files 2 and 3.

## Conclusions

We present BSmooth, a data analysis pipeline that permits precise and accurate estimates of methylation profiles with low coverage WGBS data. The pipeline starts with sequencing reads, aligns them in a bisulfite-aware fashion, compiles per-sample CpG level measurement tables, estimates methylation profiles precisely and accurately, and reports a table of DMRs taking biological variability into account. We demonstrated that our method outperforms existing methods based on Fisher's exact test. Although our comparison was limited because only two datasets appropriate for assessment were available, we expect our work to serve as a model for further assessments based on new datasets as they become available.

Finally, note that BSmooth assumes that the true methylation profile is smooth. In genomic regions where the true methylation profile is not smooth, BSmooth will still provide smooth estimates. Thus, biological events involving single CpGs might not be detected by our procedure. However, our method is well-suited for functional differences involving multiple CpGs working in conjunction.

## Materials and methods

### Datasets

The Lister data are from a WGBS experiment on the IMR90 fibroblast cell line. Six different library preparations were sequenced individually on an Illumina sequencer using up to 87 bp single-end reads and subsequently pooled to yield 25× coverage of CpGs. The Hansen data are from a WGBS experiment on three paired tumor-normal colon samples, sequenced on ABI SOLiD using 50 bp single-end reads with a CpG coverage of 4×. These data were prepared and sequenced in the laboratory of AP Feinberg. The Hansen-capture data comprise the same six samples as the Hansen data sequenced on an Illumina sequencer with up to 80 bp single reads, using a bisulfite padlock probe (BSPP) capture protocol, yielding a CpG coverage of 11× to 57× of 40,000 capture regions (one sample had substantially lower coverage than the rest, and the capture regions varied in efficiency). These data were prepared and sequenced in the laboratory of K Zhang. The Tung data are from a WGBS experiment on peripheral blood mononuclear cells from six rhesus macaque individuals, three of high social rank and three of low social rank. The data were sequenced using an Illumina sequencer with 75 bp single end reads, yielding a CpG coverage of 11× to 14×.

The Lister data were created in the following way: we obtained the raw reads from the IMR90 cell line and aligned against the hg19 genome using Merman with iterative trimming. Prior to alignment, two bases were trimmed from the start of the read and one base from the end of the read. Based on our M-bias plots, we furthermore filtered the last ten bases of every read (based on its trimmed length), when we summarized the methylation evidence. Based on the quality control plots, the flowcells marked ECKER_1062 were discarded. These data form the basis for all analysis of the Lister data in the manuscript as well as Figures S1 to S4 in Additional file 1.

In order to produce Figure S5 in Additional file 1 we obtained aligned and summarized data from the Salk Institute website [29], specifically the two files mc_imr90_r1.tar.gz and mc_imr90_r2.tar.gz. For these two files, methylation calls in non-CpG context (of which there were very few) were discarded and their stranded methylation calls were summed into calls without strand. These files were mapped against the hg18 reference genome. We converted the coordinates from hg18 to hg19 using the liftOver tool from University of California, Santa Cruz.

The Hansen WGBS data were aligned against hg19 without iterative trimming due to the short read length. Prior to alignment, we trimmed the primer base and one color from the start of the reads (this is a standard procedure before aligning colorspace reads and was not related to quality control assessment). Based on our M-bias plots we filtered 3 bp on either side of the read as part of summarizing the methylation evidence.

The Hansen-capture data were aligned using iterative trimming, without trimming any initial bases before alignment. Based on our M-bias plots we filtered the first 15 bases of each read as part of summarizing the methylation evidence.

The Tung data were aligned against rheMac 2.0 using Bismark [10]. The reads were truncated to 70 bp prior to alignment and the first three bases of each read were filtered as part of summarizing the methylation evidence. Additional detail is in [16]. The WGBS data were smoothed using the same parameters as for human data.

We obtained the preprocessed gene expression data presented in the Tung *et al*. manuscript from the journal website. TSSs were obtained from the authors (personal communication).

### Smoothing

We denote the number of reads associated with the *j*th CpG being methylated and unmethylated with *M_j _*and *U_j_*, respectively. The CpG-level summary is simply the proportion *M_j_*/*N_j_*, with *N_j_*=*M_j_*+*U_j _*the coverage for the *j*th CpG. We assume each *M_j _*follows a binomial distribution with success probability *π_j_*. The success probability represents the true proportion of cells for which the *j*th CpG is methylated in the sample being assayed. The proportion *M_j_*/*N_j _*is an unbiased estimate of π*_j _*with standard error $\sqrt{\pi_{j}\left(1-\pi_{j}\right)/N_{j}}$, and we denote ${\hat{\pi }}_{j}\equiv M_{j}/N_{j}$ the single-CpG methylation estimate of π*_j_*. We furthermore assume that π*_j _*is defined by a smoothly varying function *f *of the genomic location, that is, for location *l_j_*, π*_j_*=*f*(*l_j_*). We estimate *f *with a local-likelihood smoother [28]. We start by choosing a genomic window size *h*(*l_j_*) for each *l_j_*. The window is made large enough so that 70 CpGs are included but at least 2 kb wide. Within each genomic window we assume log[*f*(*l_j_*)/{1-*f*(*l_j_*)}] is approximated by a second degree polynomial. We assume that data follow a binomial distribution and the parameters defining the polynomial are estimated by fitting a weighted generalized linear model to the data inside the genomic window. For data points inside this window, indexed by *l_k_*, weights are inversely proportional to the standard errors of the CpG-level measurements, $\sqrt{\pi_{k}\left(1-\pi_{k}\right)/N_{k}}$, and decrease with the distance between the loci |*l_k_*-*l_j_*| according to a tricube kernel (Figure 3a,b). Note that the smoothness of our estimated profile $\hat{f}(l_{j})$ depends on genomic CpG density. We recommend users adapt the algorithm's parameters when applying it to organisms other than human.

### Identification of differentially methylated regions

To find regions exhibiting consistent differences between groups of samples, taking biological variation into account, we compute a signal-to-noise statistic similar to the *t*-test. Specifically, we denote individuals with *i *and use *X_i _*do denote group; for example, *X_i_*=0 if the *i*th sample is a control and *X_i_*=1 if a case. The number of controls is denoted *n*_1 _and the number of cases *n*_2_. We assume that the samples are biological replicates within a group. Similar to the previous section, we denote the number of reads for the *i*th sample associated with the *j*th CpG being methylated and unmethylated with *M_i,j _*and *U_i,j_*, respectively. We assume that *Y_i,j _*follows a binomial distribution with *M_i,j_*+*U_i,j _*trials and success probability π*_i,j_*, which we assume is a sample-specific smooth function of genomic location *l_j_*: π*_i,j_*=*f*_i_(*l_j_*). Furthermore, we assume that *f_i _*has the form *f_i_*(*l_j_*)=α(*l_j_*)+β(*l_j_*)*X_i_*+ε*_i,j_*. Here α(*l_j_*) represents the baseline methylation profile and β(*l_j_*) the true difference between the two groups. The latter is the function of interest, with non-zero values associated with DMRs. The ε*_i,j _*s represent biological variability with the location-dependent variance var(ε*_i,j_*)≡σ^2^(*j*) assumed to be a smooth function. Note that increasing coverage does not reduce the variability introduced by ε; for this we need to increase the number of biological replicates.

We use the smoothed methylation profiles described in the previous section as estimates for the fi, denoted ${\hat{f}}_{i}(l_{j})$. We estimate α and β as empirical averages and difference of averages: $\hat{\alpha }(l_{j})=\sum_{i}{\hat{f}}_{i}(l_{j})$ and $\hat{\beta }(l_{j})=\sum_{i:X_{i}=1}{\hat{f}}_{i}(l_{j})-\sum_{i:X_{i}=0}{\hat{f}}_{i}(l_{j})$. To estimate the smooth location-dependent standard deviation, we first compute the empirical standard deviation across the two groups. To improve precision, we used an approach similar to [30]: we floored these standard deviations at their 75th percentile. To further improve precision, we smoothed the resulting floored values using a running mean with a window size of 101. We denote this final estimate of local variation with $\hat{\sigma }(l_{j})$. We then formed signal-to-noise statistics: $t(l_{j})=\hat{\beta }(l_{j})/[\hat{\sigma }(l_{j})\sqrt{1/n_{1}+1/n_{2}}]$. To find DMRs, that is, regions for which β(*l_j_*)≠0, we defined groups of consecutive CpGs for which all *t*(*l_j_*)>*c *or *t*(*l_j_*)<-*c *with *c*>0 a cutoff selected based on the marginal empirical distribution of *t*. We adapted our algorithm so that CpGs further than 300 bp apart were not permitted to be in the same DMR.

We recommend including in the procedure only CpGs that have some coverage in most or all samples. Furthermore, we recommend filtering the set of DMRs by requiring each DMR to contain at least three CpGs, have an average β of 0.1 or greater, and have at least one CpG every 300 bp.

### Practical considerations

#### Sequencing effort

BSmooth can estimate methylation precisely with as little as 4× average coverage, but two additional points should be considered regarding sequencing depth. First, greater depth generally allows a greater fraction of CpGs to be covered with read-level measurements. Second, in addition to depth, a key concern is the length of the reads and whether the reads are paired-end reads. Longer reads and paired-end reads are more likely to align with high mapping quality, that is, a low probability of having been aligned to the wrong location. Alignments with higher mapping quality lead to higher-confidence read-level measurements.

At the time of writing, a single lane of the Illumina HiSeq 2000 instrument produces about 35 to 45 billion nucleotides of bisulfite sequencing data. After discarding low-quality alignment and bases, this results in around 19 million CpGs with a coverage of 2 or greater. If two lanes are used per sample, the increased depth results in around 23 million CpGs with a coverage of 2 or greater.

#### Non-CpG methylation

In humans, extensive non-CpG methylation has only been observed in embryonic stem cells [3]. We have not used BSmooth to study non-CpG methylation in humans, but we hypothesize it would be well suited for this purpose. Note that the alignment part of BSmooth is not affected by non-CpG methylation provided the sequencing reads are generated in nucleotide space and not colorspace. Note that there are many more Cs in the genome than CpGs; thus, analyzing these data greatly increased the memory requirements of our software. Although the current implementation does not allow this, it is a software issue that could potentially be addressed.

#### Detection limit

BSmooth assumes that the true methylation profile is smooth. In genomic regions where the true methylation profile is not smooth, BSmooth will still provide smooth estimates. Thus, biological events involving single CpGs might not be detected by our procedure. However, our procedure should still be useful if a single CpG is associated with a biological event, provided that changes in methylation of this single CpG also lead to changes in methylation of nearby CpGs. Detecting methylation changes in a single CpG without changes in nearby CpGs would need to use single-CpG estimates based on higher coverage than 4×. Such single-CpG estimates could potentially be more affected by technical biases. Note that Fisher's exact test does not account for biological variation.

### Modification of the algorithm for analysis of cancer datasets

Note that between-sample variability is larger in cancer samples [1]. If one is interested in detecting DMRs in which the cancer varies but the normal samples are consistent, then we recommend using only the normal samples to estimate σ(*j*). Cancer/normal comparisons also exhibit large blocks of hypo-methylation in cancer [1]. These blocks are much longer genomic regions than previously reported DMRs and are observed in CpG sparse genomic regions. To account for these features we adapted the DMR algorithm as described in detail in Hansen *et al*. [1] and below.

To identify large hypomethylated blocks in cancer, we changed the DMR detection algorithm in two ways: first, we changed the smoothing algorithm described above to increase the window size to include 500 CpGs of at least 40 kb wide. We also relaxed the cutoff on the signal-to-noise statistics, since many more CpGs are involved in blocks. This method - essentially the same method as used to find small scale DMRs, but using smoothing across a wider window - identifies large scale changes that are consistently different between cancer and normals. In case these large scale changes have different boundaries in different samples, this method will detect segments that are consistently different. However, in Hansen *et al*. [1] we show that the observed boundaries appear to be consistent across samples.

Once these large scale changes were identified we also modified the algorithm to identify small-scale DMRs (<10 kb) within the block regions, as described below. For all of this, we use an estimate of σ(*j*) that is only based on the three normal samples, as described above. Given the large hypo-methylated blocks in cancer, it is necessary to update the model described in the section on 'Identification of differentially methylated regions' as described above. The previous model assumes that *f_i_*(*l_j_*)=α(*l_j_*)+β(*l_j_*)*X_i_*+ε*_i,j_*. We now additionally assume that β(*l_j_*) has the form β(*l_j_*)=β_1_(*l_j_*)+β_2_(*l_j_*), and thus is composed of two components, β_1_(*l_j_*) and β_2_(*l_j_*), representing small DMRs and blocks, respectively. Note that β_2_(*l_j_*) is much more slowly varying than β_1_(*l_j_*). The signal-to-noise statistics *t*(*l_j_*), described in the section on 'Identification of differentially methylated regions', should be large (in absolute value) when either β_1 _or β_2 _are different from 0. Because β(*l_j_*) now consists of two components, the signal-to-noise statistic *t*(*l_j_*) also decomposes into two components *t*(*l_j_*)=*t*_1_(*l_j_*)+*t*_2_(*l_j_*), with the first component associated with β_1_(*l_j_*) and the second, slowly varying, component associated with β_2_(*l_j_*). In order to find small DMRs, we need to form an estimate of the second component, denoted ${\tilde{t}}_{2}(l_{j})$, and form corrected signal-to-noise statistics $t(l_{j})-{\tilde{t}}_{2}(l_{j})$. We estimate *t*_2_(*l_j_*) by identifying the slow-varying component of *t*(*l_j_*) in the following way: first we interpolate *t*(*l_j_*) to define *t*(*l*) for a general genomic location *l*. This function is evaluated at a 2 kb grid, and smoothed using a robust local likelihood model with a window size of 50 kb. This slowly varying function is then evaluated at CpG locations *l_j _*to form the estimate ${\tilde{t}}_{2}(l_{j})$. We the identify small DMRs by using corrected signal-to-noise statistics $t(l_{j})-{\tilde{t}}_{2}(l_{j})$ instead of *t*(*l_j_*), in the section on 'Identification of differentially methylated regions'.

### ROC curves and Fisher's exact test

We defined gold standard regions as follows. We consider high-coverage CpGs to be CpGs with a coverage ≥30×, and we use the pre-defined capture regions. For the first definition of positive and negative regions, we include regions for which at least two out of three cancer samples and at least two out of three normal samples have at least five high-coverage CpGs. This was done because one of the normal samples had lower coverage than the other two. For each such region we compute the average methylation in the cancer samples and the normal samples by first averaging methylation across high-coverage CpGs within a sample and then average across samples. Positives were defined as regions with difference between average cancer methylation and average normal methylation >0.25. Negatives were defined as regions for which the difference is <0.03. For the second definition, we compute the sample-specific average methylation level across the capture region using only high-coverage CpGs, and we only include regions with at least four high-coverage CpGs in each of the six samples. This was done because the Welch *t*-test requires at least three samples in each group, but it also leads to the exclusion of many regions included in the first definition, because of the single sample with lower coverage. For each region with data from all six samples, a Welch *t*-test was done on six numbers representing the average methylation across the region in each sample. Positives were such regions with an unadjusted *P*-value <1%. Negatives were such regions with an unadjusted *P*-value >25%.

We implemented a DMR finder based on Fisher's exact test, closely following the description in the supplementary material of Lister *et al*. [3]. We were able to reproduce 99% of the DMRs reported in that study. This DMR finder produces DMRs that are at least 2 kb long, containing at least 10 CpGs that are differentially methylated according to Fisher's exact test. In addition, every 1 kb subregion contains at least four such CpGs.

### Software

BSmooth is open source software [31].

## Abbreviations

DMR: differentially methylated region; FDR: false discovery rate; ROC: receiver operating characteristic; TSS: transcription start site; WGBS: whole-genome bisulfite sequencing.

## Competing interests

The authors declare that they have no competing interests.

## Authors' contributions

KDH and RAI designed the smoothing method, and KDH implemented it. BL designed and implemented the alignment methods. All authors read and approved the final manuscript for publication.

## Supplementary Material

Additional file 1 **Additional figures**. A PDF file containing Figures S1 to S5. Click here for file

Additional file 2 **Alignment code**. Click here for file

Additional file 3 **Data analysis code**. Click here for file
