# Supplementary material for: BSmooth: from whole genome bisulfite sequencing reads to differentially methylated regions
Source: Genome Biol. 2012 Oct 3;13(10):R83. doi: 10.1186/gb-2012-13-10-r83 (PMC3491411; doi:10.1186/gb-2012-13-10-r83)
Supplement: Additional file 1 — Additional figures. A PDF file containing Figures S1 to S5. [file gb-2012-13-10-r83-S1.pdf]

Additional File 1
Additional figures
for
BSmooth: from whole genome bisulfite sequencing reads to differentially methylated regions

Kasper D. Hansen Benjamin Langmead Rafael A. Irizarry

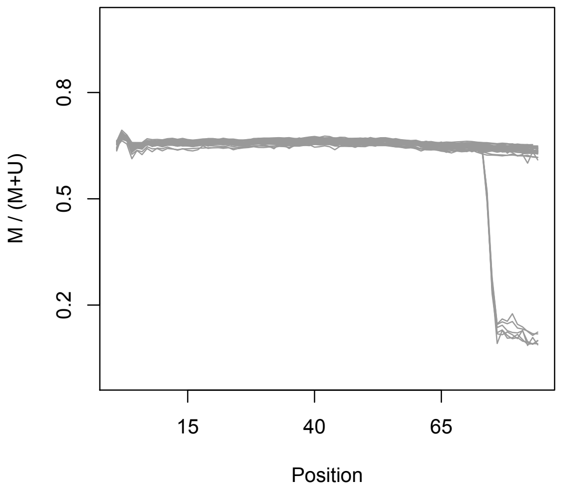

Figure S1. M-bias plot of the Lister data. The data has been aligned using iterative trimming and each line corresponds to a separate flowcell worth of data. The M-bias plot has not been stratified by read length, unlike Figure 1b. Comparing these two figures, it is clear that not stratifying by read length may hide biases at the end of the reads. In addition, we observe 5 flowcells with very biased methylation values for their last 10bp.

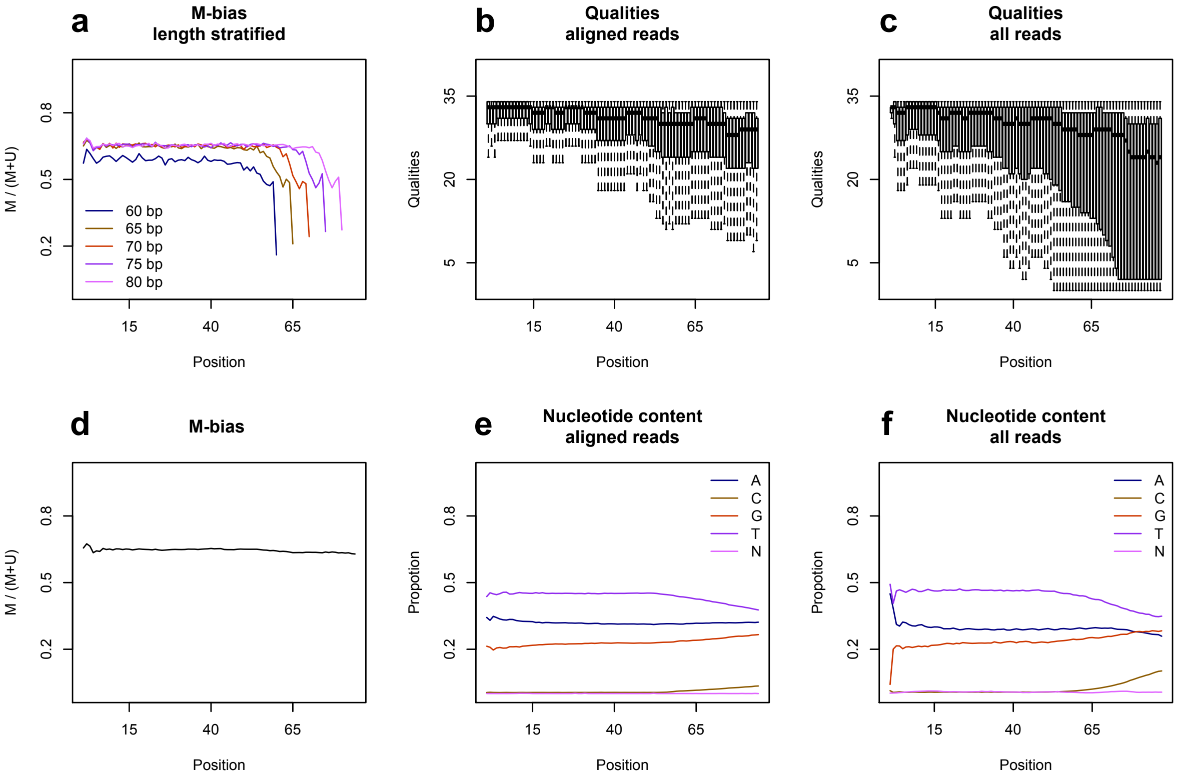

Figure S2. A suite of quality control plots for a single flowcell, "R1B_DARWIN_4011". (a) A length-stratified M-bias plot, like Figure 1b, but for a single flowcell. (b) Boxplots of position-specific base-call quality scores for aligned reads. (c) As (b), but for all reads. (d) An M-bias plot that is not stratified by read length. (e) Nucleotide content per position for aligned reads. (f) as (e) but for all reads. This depicts a well-behaved flowcell with some methylation bias at the end of each (trimmed) read. This bias is only visible in the length-stratified M-bias plot.

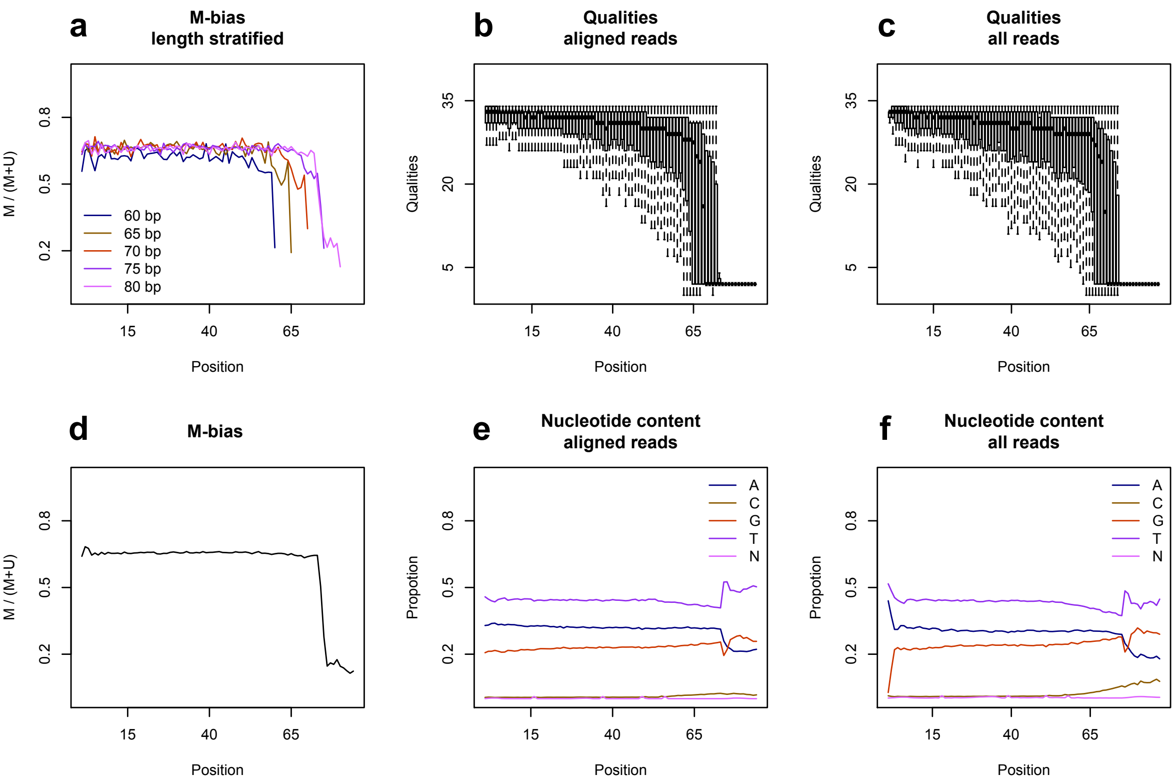

Figure S3. A suite of quality control plots for a single flowcell, "R1B_ECKER_1062". Like Figure S2, but for a more problematic flowcell based on the same library preparation. In this flowcell, the last cycles (positions 70 and onward) have very low quality values and a large methylation bias. This does not seem to affect the shorter reads in (a), although they still exhibit methylation bias at the last bases, as in Figure S2.

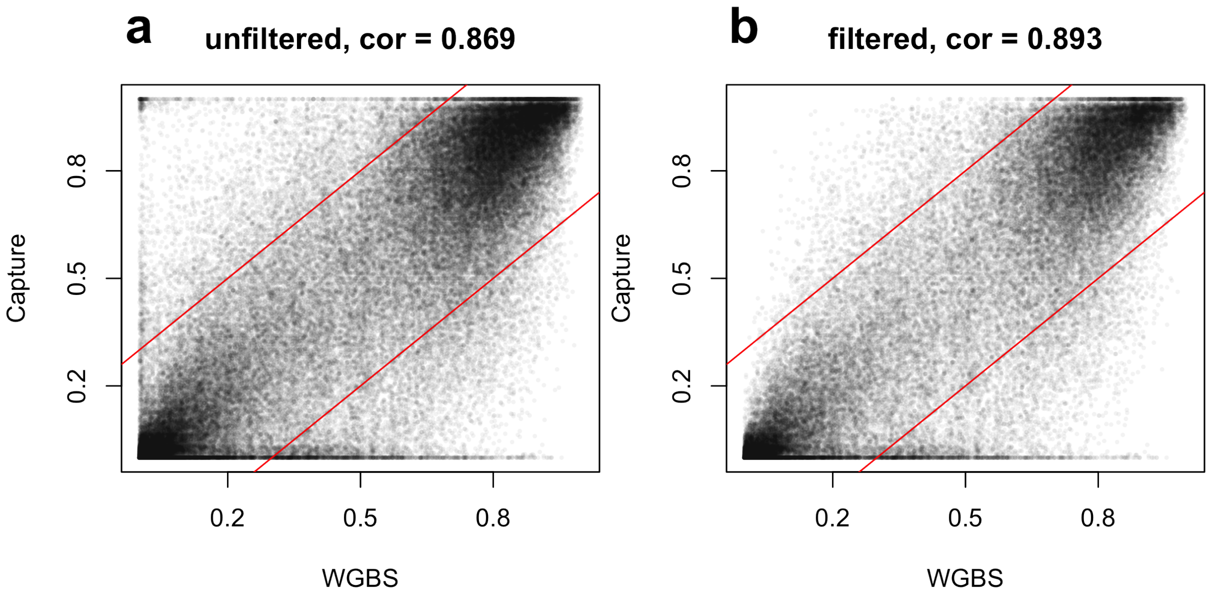

Figure S4. Comparison of Hansen and the Hansen-capture data with and without M-bias filtering the capture data. Depicted are data from the sample “normal 2”, from both the Hansen data (whole-genome bisulfite sequencing) and Hansen-capture (capture bisulfite sequencing), for CpGs for which the capture data greater than 30x coverage. (a) A scatterplot of single-base methylation estimates from the capture data against smoothed methylation estimates from WGBS. Note the many discrepant points in the upper left corner. (b) Based on the M-bias plots (Fig. 2c) filtering was performed on the capture bisulfite data. This removes the discrepant points visible in (a) where WGBS showed a methylation level around 0 and capture bisulfite sequencing showed a methylation level around 1.

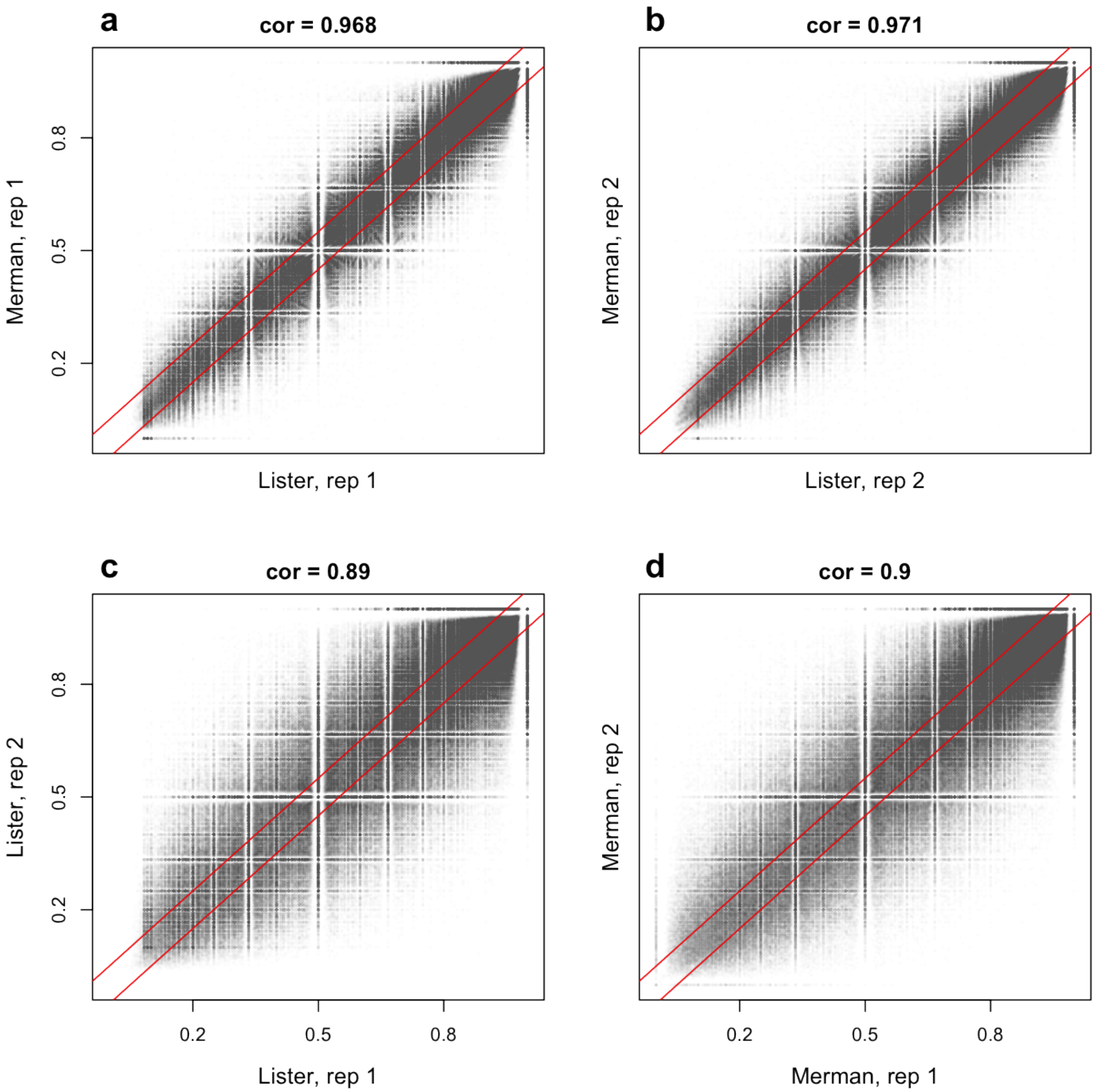

Figure S5. Comparison of single resolution methylation calls using different alignment strategies. The Lister data was aligned using "Merman" aligner described in this work and compared to the results from the original publication. Each of the two separate extractions were processed individually and labeled "Merman, rep 1,2" and "Lister, rep 1,2". We selected all CpGs that had a coverage > 10x in both replicates and both alignment strategies (n=9.7M) and estimated single base methylation levels as $M/(M+U)$. Depicted are scatterplots of these methylation estimates, using a subsample of 10^6 CpGs selected at random. Alpha-blending is used to estimate the local density of points and the red lines are $y = x \pm 0.05$. We observe that the variation introduced by using the two different alignment and filtering strategies on the same extraction is much smaller than the variation between the two different extractions from the same cell line using a single alignment strategy.
