# Supplementary material for: BSmooth: from whole genome bisulfite sequencing reads to differentially methylated regions
Source: Genome Biol. 2012 Oct 3;13(10):R83. doi: 10.1186/gb-2012-13-10-r83 (PMC3491411; doi:10.1186/gb-2012-13-10-r83)
Supplement: Additional file 3 — Data analysis code. [file gb-2012-13-10-r83-S3.gz › bsseq/inst/doc/bsseq_analysis.pdf]

Analyzing WGBS with the bsseq package

Kasper Daniel Hansen

Modified: June 26, 2011. Compiled: June 26, 2012

Introduction

This document discusses the ins and outs of an analysis of a whole-genome shotgun bisulfite sequencing (WGBS) dataset, using the BSmooth algorithm, which was first used in [1] and more formally presented and evaluated in [2]. The intention with the document is to focus on analysis-related tasks and questions. Basic usage of the `bsseq` package is covered in "The bsseq user's guide". It may be useful to consult the user's guide while reading this analysis guide.

The analysis covered in this guide is too big to run on the Bioconductor build servers (since they have a time limit of 5 minutes). But the analysis is certainly not too big to run on a modest computer system, and it should not take prohibitively long to do so (less than 1 hour for sure). FIXME: better time estimate.

In this vignette we analyze chromosome 21 and 22 from [1]. This is primary data from 3 patients with colon cancer. For each patient we have normal colon tissue as well as cancer colon. The samples were run on ABI SOLiD and we generated 50bp single-end reads. The reads were aligned using the Merman aligner in the BSmooth suite (http://www.rafalab.org/bsmooth). See the primary publication for more details [1].

This data is contained in the `bsseqData`

```
> library(bsseq)
> library(bsseqData)
```

The `bsseqData` contains a script, `inst/script/create_BS.cancer.R`, describing how this data is created from the Merman alignment output (also contained in the package). Note that the current version of the BSmooth pipeline uses a slightly different alignment output format.

The following object contains the unsmoothed "raw" summarized alignment data.

```
> BS.cancer.ex
```

```
An object of type 'BSseq' with
  958541 methylation loci
  6 samples
has not been smoothed
```

```
> pData(BS.cancer.ex)
```

```
     Type  Pair
C1 cancer pair1
C2 cancer pair2
C3 cancer pair3
N1 normal pair1
N2 normal pair2
N3 normal pair3
```

Smoothing

The first step of the analysis is to smooth the data

```
> BS.cancer.ex.fit <- BSmooth(BS.cancer.ex, mc.cores = 6, verbose = TRUE)
```

For ease of use, the `bsseqData` includes the result of this command:

```
> data(BS.cancer.ex.fit)
> BS.cancer.ex.fit
```

```
An object of type 'BSseq' with
  958541 methylation loci
  6 samples
has been smoothed with
  BSmooth (ns = 70, h = 1000, maxGap = 100000000)
```

This step uses parallelization where each sample is run on a separate core using `mclapply` from the `parallel` package. This form of parallelization is built into `bsseq`, and (as written) requires access to a machine with 6 cores and enough RAM. The smoothing step is being done completely independently on each sample, so if you have a lot of samples (or other circumstances), an alternative is to split the computations manually. A later subsection shows some example code for doing that.

Let us discuss coverage and representation. The `BS.cancer.ex` object contains all annotated CpGs on human chromosome 21 and 22, whether or not there is actual data. Since we have multiple samples, we can roughly divide the genome into 3 categories: CpGs where all samples have data, CpGs where none of the samples have data and CpGs where some, but not all, of the samples have data. Examining the object at hand, we get

```
> ## The average coverage of CpGs on the two chromosomes
> round(colMeans(getCoverage(BS.cancer.ex)), 1)
```

```
 C1  C2  C3  N1  N2  N3
3.5 4.2 3.7 4.0 4.3 3.9
```

```
> ## Number of CpGs in two chromosomes
> length(BS.cancer.ex)
```

```
[1] 958541
```

```
> ## Number of CpGs which are covered by at least 1 read in all 6 samples
> sum(rowSums(getCoverage(BS.cancer.ex) >= 1) == 6)
```

```
[1] 572628
```

```
> ## Number of CpGs with 0 coverage in all samples
> sum(rowSums(getCoverage(BS.cancer.ex)) == 0)
```

```
[1] 136019
```

The CpG coverage is roughly 4x, so we would expect many zero coverage CpGs by chance. although that should not necessarily occur in all 6 samples at the same CpG. If we assume that coverage genome-wide is Poisson distributed with a parameter (lambda) of 4, we would expect

```
> logp <- ppois(0, lambda = 4, lower.tail = FALSE, log.p = TRUE)
> round(1 - exp(6 * logp), 3)
```

```
[1] 0.105
```

of the CpGs to have at least one sample with zero coverage.

There are roughly 130k CpGs with no data at all in any of the 6 samples. This can happen either because of chance (although that is unlikely) or because the CpG is unmappable. Since we are dealing with bisulfite converted reads, the unmappable portion of the genome is greater than with normal DNA-sequencing. For this experiment we only used 50bp single-end reads (in our experience using 100bp paired-end reads greatly increases the mappable percentage of the genome). These CpGs (with zero coverage in all samples) are in some sense easy to deal with: one should of course be careful drawing conclusions about CpGs with no data.

We have roughly $959 - 573 - 136 = 250$k CpGs where some (but not all) of the samples have zero coverage, and these are in some sense harder to deal with. Since we have very low coverage to begin with, it may happen just by chance that a single sample have zero coverage, and it may be too restrictive to just exclude these CpGs from an analysis.

Smoothing is done separately for each sample, only using the data where the coverage (for that sample) is non-zero. This estimates a genome-wide methylation profile, which is then

evaluated in all CpGs in the "BSseq" object. As a result, after smoothing, every CpG in the object has an estimated methylation value. This is very nice for the situation where you want to compare a single CpG across multiple samples, but one or two of the samples have zero coverage by chance. But note that these smoothed methylation profiles makes less sense in the parts of the genome where there are no covered CpGs nearby. We fix this by removing these CpGs after smoothing, see below.

Other arguments to the `BSmooth` function are `mc.cores`, `mc.preschedule`, `parallelBy` which controls the parallelization built into the function as well as `ns`, `h`, `maxGap` which controls the smoothing. `ns` is the minimum number of CpGs contained in each window, `h` is half the minimum window with (the actual window width is either 2 times `h` or wide enough to contain `ns` covered CpGs, whichever is greater). Note that the window width is different at each position in the genome and may also be different for different samples at the same position, since it depends on how many nearby CpGs with non-zero coverage. Per default, a smoothing cluster is a whole chromosome. By "cluster" we mean a set of CpGs which are processed together. This means that even if there is a large distance between two CpGs, we borrow strength between them. By setting `maxGap` this can be prevented since the argument describes the longest distance between two CpGs before a cluster is broken up into two clusters.

Manually splitting the smoothing computation

An example, only showing sample 1 and 2 for brevity, is (this example is not being run):

```
> ## Split datag
> BS1 <- BS.cancer.ex[, 1]
> save(BS1, file = "BS1.rda")
> BS2 <- BS.cancer.ex[, 2]
> save(BS1, file = "BS1.rda")
> ## done splitting
>
> ## Do the following on each node
>
> ## node 1
> load("BS1.rda")
> BS1.fit <- BSmooth(BS1)
> save(BS1.fit)
> save(BS1.fit, file = "BS1.fit.rda")
> ## done node 1
>
> ## node 2
> load("BS2.rda")
> BS2.fit <- BSmooth(BS2)
```

```
> save(BS2.fit, file = "BS2.fit.rda")
> ## done node 2
>
> ## join; in a new R session
> load("BS1.fit.rda")
> load("BS2.fit.rda")
> BS.fit <- combine(BS1.fit, BS2.fit)
```

This still requires that you have one node with enough RAM to hold all samples in memory.

Computing t-statistics

Before computing t-statistics, we will remove CpGs with little or no coverage. If this is not done, you may find many DMRs in areas of the genome with very little coverage, which are most likely false positives. It is open to personal preferences exactly which CpGs to remove, but for this analysis we will only keep CpGs where at least 2 cancer samples and at least 2 normal samples have at least 2x in coverage. For readability, we store the coverage in a separate matrix (this is just due to line breaks in Sweave)

```
> BS.cov <- getCoverage(BS.cancer.ex.fit)
> keepLoci <- which(rowSums(BS.cov[, c("C1", "C2", "C3")] >= 2) >= 2 &
+                   rowSums(BS.cov[, c("N1", "N2", "N3")] >= 2) >= 2)
> length(keepLoci)
```

```
[1] 597371
```

```
> BS.cancer.ex.fit <- BS.cancer.ex.fit[keepLoci,]
```

We are now ready to compute t-statistics, by

```
> BS.cancer.tstat <- BSmooth.tstat(BS.cancer.ex.fit,
+                                  group1 = c("C1", "C2", "C3"),
+                                  group2 = c("N1", "N2", "N3"),
+                                  estimate.var = "group2",
+                                  local.correct = TRUE,
+                                  verbose = TRUE)
```

```
preprocessing ... done in 1.0 sec
computing stats within groups ... done in 0.4 sec
computing stats across groups ... done in 1.8 sec
```

```
> BS.cancer.tstat
```

```
An object of type 'BSseqTstat' with
  597371 methylation loci
```

```
based on smoothed data:
  BSmooth (ns = 70, h = 1000, maxGap = 100000000)
with parameters
  BSmooth.tstat (local.correct = TRUE, maxGap = 100000000)
```

The arguments to `BSmooth.tstat` are simple. `group1` and `group2` contain the sample names of the two groups being compared (it is always group1 - group2), and indices may be used instead of sample names. `estimate.var` describes which samples are being used to estimate the variability. Because this is a cancer dataset, and cancer have higher variability than normals, we only use the normal samples to estimate the variability. Other choices of `estimate.var` are `same` (assume same variability in each group) and `paired` (do a paired t-test). The argument `local.correct` describes whether we should use a large-scale (low-frequency) mean correction. This is especially important in cancer where we have found many large-scale methylation differences between cancer and normals.

We can look at the marginal distribution of the t-statistic by

```
> plot(BS.cancer.tstat)
```

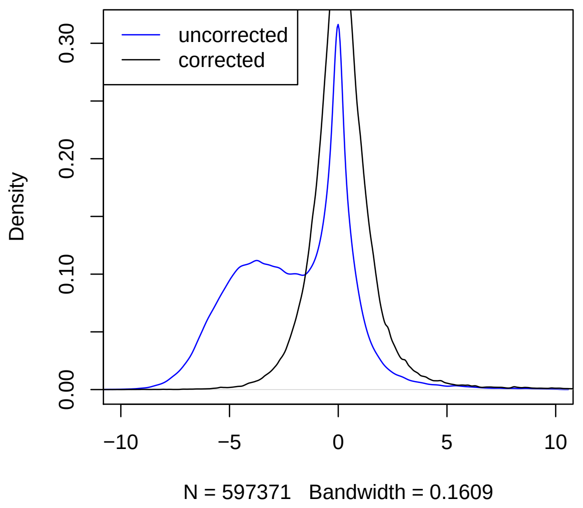

The "blocks" of hypomethylation are clearly visible in the marginal distribution of the uncorrected t-statistics.

Even in comparisons where we do not observe these large-scale methylation differences, it often improves the marginal distribution of the t-statistics to locally correct them ("improves" in the sense of making them more symmetric).

Finding DMRs

Once t-statistics have been computed, we can compute differentially methylated regions (DMRs) by thresholding the t-statistics. Here we use a cutoff of 4.6, which was chosen by looking at the quantiles of the t-statistics (for the entire genome).

```
> dmrs0 <- dmrFinder(BS.cancer.tstat, cutoff = c(-4.6, 4.6))
```

```
segmenting
splitting
creating dmr data.frame
```

```
> dmrs <- subset(dmrs0, n >= 3 & abs(meanDiff) >= 0.1)
> nrow(dmrs)
```

```
[1] 373
```

```
> head(dmrs, n = 3)
```

```
     chr    start      end idxStart idxEnd cluster   n  meanDiff
14 chr21 28215373 28219673    57140  57375   15140 236 0.3423310
21 chr21 32929738 32932526    79950  80169   20172 220 0.3616328
26 chr21 34441912 34444680    92256  92480   21568 225 0.3093227
   group1.mean group2.mean   tstat.sd areaStat  maxStat direction
14   0.3870996  0.04476853 0.04503204 2623.165 13.50173     hyper
21   0.4174597  0.05582693 0.04492697 2326.016 12.16182     hyper
26   0.3406376  0.03131488 0.04604162 2156.520 12.39799     hyper
   width invdensity
14  4301   18.22458
21  2789   12.67727
26  2769   12.30667
```

Here, we filter out DMRs that do not have at least 3 CpGs in them and at least a mean difference (across the DMR) in methylation between normal and cancers of at least 0.1. While the exact values of these two filters can be debated, it is surely a good idea to use something like this.

Other arguments to `dmrFinder` are `qcutoff` which chooses a quantile-based cutoff (for example `qcutoff = c(0.01, 0.99)`) and `maxGap` which makes sure that a DMR is being split if there are two CpGs with more than `maxGap` between them (default of 300bp).

We rank DMRs by the column `areaStat` which is the sum of the t-statistics in each CpG. This is kind of the area of the DMR, except that it is weighted by the number of CpGs and not by genomic length. This is currently the best statistic we know, although it is far from perfect (we would like to do something better).

Plotting

It is *always* a good idea to look at the DMRs. One way of encoding standard plotting parameters like `col`, `lty`, and `lwd` is to add columns to the `pData`, like

```
> pData <- pData(BS.cancer.ex.fit)
> pData$col <- rep(c("red", "blue"), each = 3)
> pData(BS.cancer.ex.fit) <- pData
```

Once this is setup, we can plot a single DMR like

```
> plotRegion(BS.cancer.ex.fit, dmrs[1,], extend = 5000, addRegions = dmrs)
```

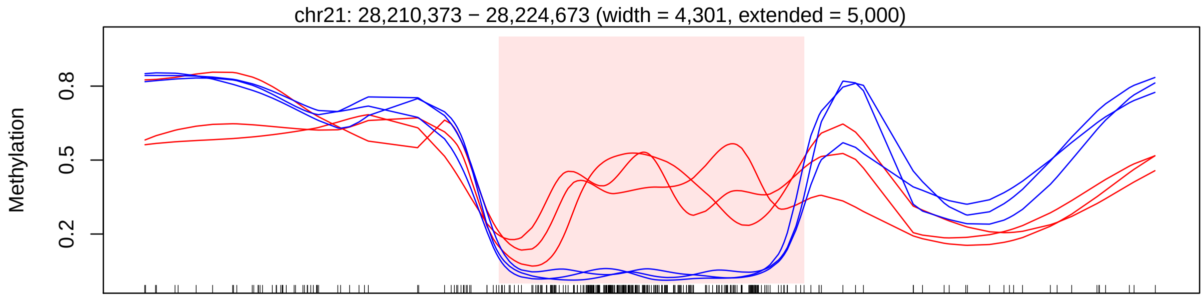

`extend` tells us how many bp to extend to either side of the plotting region. `addRegions` is a `"data.frame"` or `GRanges` listing additional regions that should be highlighted.

Typically, we plot hundreds of DMRs in a single PDF file and use external tools to look at them. For this purpose, `plotManyRegions` is very useful since it is much faster than plotting individual DMRs with `plotRegion`. An example (not run) is

```
> pdf(file = "dmrs_top200.pdf", width = 10, height = 5)
> plotManyRegions(BS.cancer.ex.fit, dmrs[1:200,], extend = 5000,
+                 addRegions = dmrs)
> dev.off()
```

which plots the top200.

Question and answers

1. The BSmooth algorithm is supposed to give smooth methylation estimates. Yet, when I plot the smoothed values, I see jagged lines, which do not look smooth to me.

We estimate a genome-wide methylation profile that is a smooth function of the genomic position. However, this profile is not stored in the "BSseq" objects. Instead, we evaluate this smooth profile in the methylation loci in the object. An example (made-up values) is

```
pos  meth
1     0.1
3     0.1
5     0.1
200   0.6
203   0.6
205   0.6
```

For plotting we do linear interpolation between this points. The end result is that the methylation profile may appear jagged especially if there is a "big" distance between two CpGs (between pos 5 and 200 above). If we wanted to plot truly smooth profiles we would have to store the methylation profile evaluated at a regular grid across the genome. This takes up a lot of space and would add complications to the internal data structures.

References

[1] Kasper D Hansen, Winston Timp, Hector Corrada Bravo, Sarven Sabunciyan, Benjamin Langmead, Oliver G. McDonald, Bo Wen, Hao Wu, Yun Liu, Dihn Diep, Eirikur Briem, Kun Zhang, Rafael A Irizarry, and Andrew P Feinberg. Generalized Loss of Stability of Epigenetic Domains Across Cancer Types. *Nature Genetics*, 43(8):768–775, 2011. doi:10.1038/ng.865, PMID:21706001.

[2] Kasper D Hansen, Benjamin Langmead, and Rafael A Irizarry. BSmooth: from whole genome bisulfite sequencing reads to differentially methylated regions. *Submitted*, 2012.

SessionInfo

- R version 2.15.1 Patched (2012-06-22 r59607), `x86_64-apple-darwin11.4.0`
- Locale: `en_US.utf-8/en_US.utf-8/en_US.utf-8/C/en_US.utf-8/en_US.utf-8`
- Base packages: base, datasets, graphics, grDevices, methods, parallel, stats, utils
- Other packages: BiocGenerics 0.2.0, bsseq 0.3.6, bsseqData 0.1.0, GenomicRanges 1.8.6, IRanges 1.14.3, matrixStats 0.5.0
- Loaded via a namespace (and not attached): Biobase 2.16.0, colorspace 1.1-1, dichromat 1.2-4, grid 2.15.1, labeling 0.1, lattice 0.20-6, locfit 1.5-8, munsell 0.3,

plyr 1.7.1, R.methodsS3 1.2.2, RColorBrewer 1.0-5, scales 0.2.1, stats4 2.15.1, stringr 0.6, tools 2.15.1
